# Exploring therapeutic aspects and prognosis of Moroccan patients with vaginal cancer: a retrospective cohort study at the University Hospital of Fez

**DOI:** 10.11604/pamj.2025.50.48.29059

**Published:** 2025-02-11

**Authors:** Wissal Hassani, Ghita Chrifi Alaoui, Kaoutar Soussy, Farhane Fatim-Zahra, Zenab Alami, Bouhafa Touria

**Affiliations:** 1 Radiotherapy Department, Hassan II University Hospital, Fez, Morocco

**Keywords:** Vaginal cancer, human papillomavirus infection, radiotherapy, multimodality

## Abstract

Primary vaginal cancer is rare, making up 1% to 2% of all female reproductive tract cancer. In Morocco, human papillomavirus (HPV) infection is the main factor. Given its low incidence, there is no consensus on the appropriate management of this cancer. The aim of our study was to assess the global management of vaginal cancer in the Radiotherapy Department, at Hassan II University Hospital, Fez, Morocco. We conducted a retrospective study of vaginal cancer cases treated at the radiotherapy department of Hassan II University Hospital between January 2012 and December 2022. Twenty-five cases of vaginal cancer were identified during the study period. In our study series, the median age was 62 years old with a range from 34 to 81 years. The average diagnostic delay was 12 months, 88% were multiparous and the notion of risky sexual behavior has been affirmed by none of them. Out of the patients we observed, 21 had squamous cell carcinoma of the vagina, one adenocarcinoma, and 3 cases of melanoma. The primary mode of treatment for the majority of our patients was concomitant radio chemotherapy, which was administered to 21 patients. Surgery was not performed on any of the patients. In the absence of consensus in the literature on the optimal treatment, concomitant radio chemotherapy remains the standard treatment for locally advanced vaginal cancer. Currently, interests are focused on the role of HPV in the genesis of vaginal cancer and on the HPV vaccine to prevent virus-induced lesions. In fact, the emphasis should be on primary prevention with prophylactic HPV vaccination.

## Introduction

Vaginal cancer is a rare malignancy that develops in the vagina and does not have any clinical or histologic evidence of cervical or vulvar cancer, or a prior history of these cancers within the last five years [1]. Primary vaginal cancer accounts for only 1% - 2% of all female genital tract malignancies and 10% of all vaginal malignant neoplasms, with an average age of diagnosis of 60 years [2,3]. The most common type of vaginal cancer is squamous cell carcinoma, followed by adenocarcinomas while the remaining cases include rare instances of melanoma, lymphoma, and sarcoma [4,5]. In addition, the vagina can be the site of secondary metastatic lesions, which require management similar to that of primary cancer [1]. In Morocco, the primary risk factor for vaginal cancer is the human papillomavirus (HPV) [6].

Due to its low incidence, there is no consensus on the appropriate management of this cancer. The management of vaginal cancer must be individualized based on the tumor's location, size, clinical stage, comorbidities, and the patient's general condition. Treatment options include surgery, chemotherapy, and radiotherapy, including brachytherapy. Radiotherapy can be an alternative to surgery, particularly in cases where surgery is not a feasible option due to an esthetic reasons [7].

Thus, we sought to evaluate the overall management of vaginal cancer in a low-middle-income country, by analyzing the cases collected in our department and assessing the clinical, diagnostic, and therapeutic aspects of this pathology.

## Methods

**Study design and setting:** we performed a retrospective descriptive analysis on patients who were diagnosed with vaginal cancer over a ten-year period from January 2012 to December 2022. The study was conducted at the Radiotherapy Department of Hassan II University Hospital in Fes, which serves a vast central region of Morocco. All patients diagnosed with vaginal cancer during this time were included, and their medical records were examined. The follow-up medical records were used to evaluate the outcome and survival of these patients.

**Study population:** we included for this study all female patients diagnosed with non-metastatic primary vaginal cancer that were treated at the radiotherapy department of the Hassan II Hospital in Fes. The study eligibility criteria were 18 years of age or older with a histological diagnosis of vaginal cancer. Patients with associated cervical cancer were excluded from the study. All patients underwent a gynecological examination with a cervico-vaginal smear. No sample size estimation was done due to the retrospective design of this study.

**Data collection:** patient identification was accomplished via the radiation therapy department's hospitalization register. Data were collected from HOSIX electronic data capture tools hosted at Hassan II University Hospital of Fez, individual patient paper records, and ARIA software treatment records. Excel 2016 software was used to input and analyze the data based on various parameters of an exploitation form. The exploitation form was developed to obtain epidemiological, clinical, histological, therapeutic, and prognostic data through bibliographic research. Variables collected included history, demographic information, diagnostic parameters, FIGO stage, therapeutic protocol, and evolution.

**Definitions:** the non-metastatic disease was defined by the absence of metastases on the thoraco-abdomino-pelvic computed tomography (CT) scan. Overall survival was calculated from diagnosis to last follow-up or date of death, toxicity was graded using the fourth edition of the Common Terminology Criteria for Adverse Events (CTCAE). In our study, we adopted the TNM 2017 classification.

**Statistical analysis:** the statistical analysis was conducted using Excel 2016 software. Descriptive statistics were used to summarize baseline patient characteristics, with qualitative variables expressed as numbers and percentages and quantitative variables expressed as means ± standard deviations (SD). Categorical data were summarized using frequencies and percentages, while numerical data were summarized using medians and interquartile ranges or means and standard deviations depending on the distribution of the variables.

**Ethical considerations:** given that the study was a retrospective observational non-interventional analysis, written consent was not deemed necessary. The work was conducted with due regard for the principles of anonymity. The ethical committee of CHU Hassan II in Fes granted ethical approval for the study.

## Results

**General characteristics:** between January 2012 and December 2022, 25 women received vaginal cancer screening at Hassan II University Hospital's Radiotherapy Department. The median age of the patients was 62 years, ranging from 34 to 81 years. Among the patients, 23 (92%) were classified as low socioeconomic status, with 64% (n=16) reporting passive smoking and only one reporting active smoking. Hormonal evaluation revealed that 68% (n=17) of the patients were menopausal at the time of diagnosis, with an average age of 50 years for menopause. None of the patients were nulliparous, while 22 (88%) were multiparous. Although none reported engaging in risky sexual behavior, 22 (88%) had a history of recurrent genital infections.

**Clinical and paraclinical presentations:** the diagnostic delay, defined as the duration between the onset of symptoms and the first consultation, ranged from 2 months to 3 years, with an average of 12 months. Leucorrhea was the most common reason for consultation, accounting for 84% of cases (n=21). Metrorrhagia and pelvic pain were also prevalent symptoms, observed in 64% (n=16) of cases, while irritative lower urinary tract symptoms such as pollakiuria and hematuria were reported in 44% (n=11) of patients. Alterations in the general condition were noted in 12% (n=3) of cases.

**Figure 1:**
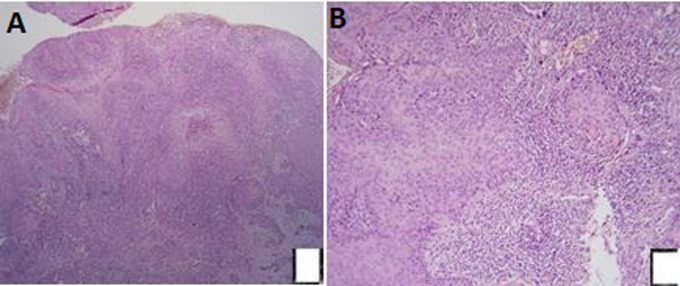
(A,B) squamous cell carcinoma of the vagina: clumps of atypical squamous cells with keratinizations (HES X100)

The most frequently observed tumors in this study were ulcerative budding tumors, accounting for 56% (n=14) of cases. The location involving the entire posterior wall was the most frequent (28%), while other locations such as the entire anterior wall, 2/3 of the inferior of the posterior or anterior wall, the upper 1/3 of the anterior wall, and all of the vagina were equally distributed, representing 14.4% of cases each. All patients underwent a systematic biopsy, which confirmed the diagnosis. Squamous cell carcinoma was the most commonly observed histological type (84%, n=21), one case of adenocarcinoma and 3 cases of melanoma were also reported (Figure 1).

**Figure 2:**
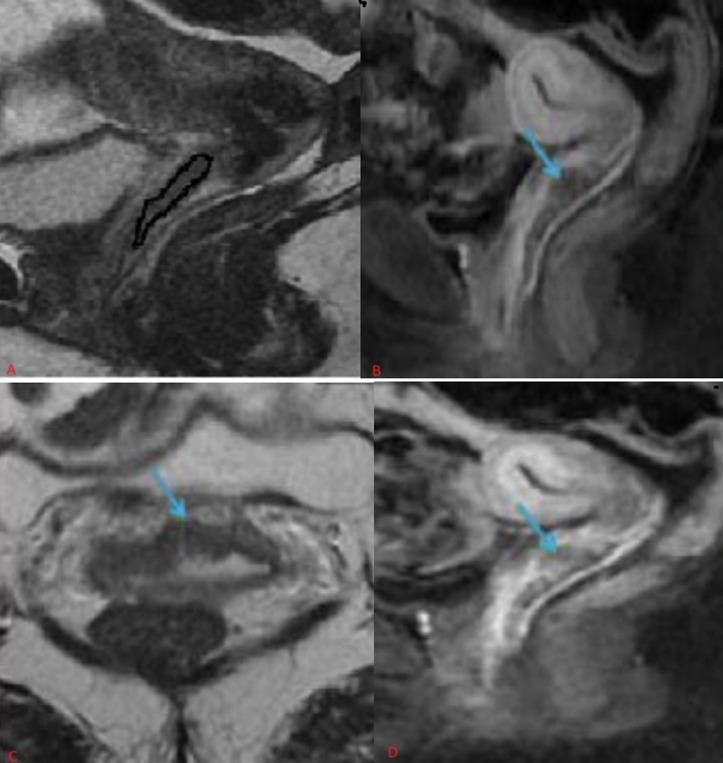
(A,B,C,D) thickening of the anterior vaginal wall is so intense at the myometrium; relative hyper signal compared to the cervical stroma (circled in black) which increases slightly and gradually after injection of phosphatidyl choline (PDC) (blue arrow), this is a lesion confined to the vagina (stage I FIGO) with distal interruption of the hypo signal of the vaginal muscularis without extension to the intervesico-vaginal fatty space

Due to the socio-economic constraints of our patient population, magnetic resonance imaging was only performed for five patients (Figure 2). No secondary locations were detected during the initial examination. Colposcopy was required for two of our patients, while the rest of the extension assessment involved a thoracoabdominopelvic CT scan, which did not reveal any secondary locations. The 3 patients with vaginal melanoma underwent cerebral magnetic resonance imaging (MRI), which did not detect any secondary locations. Metastases to the bone are rare in vaginal tumors, however, they are common in vaginal tumors with unfavorable histology, and can be detected using a bone scan. In our series, no cases of bone metastases were reported.

Tumor stage analysis revealed that T3 stage was the most prevalent form, observed in 80% of patients (n=20), followed by T4 stage in 16% of patients (n=4), and T1 stage in only one patient. Lymph node analysis showed N0 stage to be the most common stage in 88% of patients (n=22), followed by N1 stage in 8% of patients (n=2). A standard biological assessment was carried out systematically for all patients to establish a pre-therapeutic and tolerance assessment, including blood grouping, complete blood count, electrolyte balance, and renal function, as well as a coagulation assessment.

**Management and outcome:** all patient files were discussed in a multidisciplinary meeting. Surgery was not indicated in any of the patients due to locally advanced stage, advanced age, and comorbidities in one patient. All patients received three-dimensional conformal radiotherapy, with a total dose of 46 grays. The radiotherapy was indicated on the entire pelvis and included inguinal lymph node areas in 5 patients. A boost on the vagina was subsequently prescribed. It was performed by external radiotherapy in 84% of patients (n=21) at a dose of 24 grays. In 16% (n=4), it was performed by high-dose-rate (HDR) contact brachytherapy at the dose of 7 grays*3. Eighty-four percent (n=21) of our patients were treated with concurrent radio chemotherapy. The concurrent chemotherapy consisted of weekly cisplatin cycles at a dose of 40 mg/m^2^. Sixteen percent (n=4) received exclusive radiotherapy.

All our patients presented with acute toxicity with diarrhea, cystitis, and rectitis grade 1, which were treated symptomatically and resolved satisfactorily. No grade 2 or higher toxicity was observed. Late complications were observed in 88% of patients (n=21) in the form of vaginal synechiae, with 2 cases of total synechiae. Additionally, 2 cases of vesicovaginal fistulas were reported.

All our patients benefited from post-therapeutic follow-up with a first consultation 6 months after the end of treatment and then quarterly. A gynecological examination was performed at each consultation and an MRI after 3 months. After a mean follow-up of 65.7 months, we noted: that complete remission was maintained in 56% of our patients (n=14), stability in 16% (n=4), metastatic progression in 20% (n=5), 1 patient died from septic shock, and 1 patient from cardiorespiratory arrest.

## Discussion

The purpose of this retrospective observational monocentric study was to examine the clinical, diagnostic, and therapeutic aspects of vaginal cancer and assess its overall management by analyzing the cases collected in our department. Twenty-five women were screened for vaginal cancer between 2012 and 2022. Most patients were low-income, menopausal, multiparous, and had a history of genital infections. The diagnosis was delayed, with leucorrhea being the most common symptom. Squamous cell carcinoma was the most common type of cancer, and radiotherapy with chemotherapy was the primary treatment. Complications included acute toxicity, vaginal synechiae, and vesicovaginal fistulas. Fifty-two percent of patients achieved complete remission, while 21% had metastatic progression. The study underscores the importance of early detection and improved screening strategies.

Vaginal cancer is rare, accounting for only 1% - 2% of female genital tract malignancies and 10% of all vaginal malignant neoplasms [1,2]. It mainly affects women aged 65 and older, with a notable peak in incidence occurring between 60 and 70 years of age. However, there is presently a trend towards a younger affected population due to the involvement of the Human Papillomavirus. In our research, the median age observed was 60 years old [5]. Limited epidemiological research has been dedicated to exploring vaginal cancer. Among the identified risk factors for this condition are the Human Papillomavirus (HPV), which plays a crucial role in the etiology of a majority of squamous cell cancers in the vagina, along with smoking [8]. In Morocco, HPV infection poses a significant public health concern, as a considerable proportion of women exhibit HPV infections and manifest precancerous lesions [9]. In our study, women were not screened for HPV infection due to socioeconomic constraints. None of the participants were active smokers; however, a substantial number of them were exposed to passive smoking.

About 70% of vaginal cancers are squamous cell carcinomas (SCCs). The cancers begin in the squamous cells that make up the epithelial lining of the vagina. About 15 of every 100 cases of vaginal cancer are adenocarcinomas. The usual type of vaginal adenocarcinoma typically develops in women older than 50 [8]. Vaginal cancer arises more often in the upper third of the vagina but can also originate in the middle or lower third; a fairly even distribution of lesions is evident, arising on the anterior, posterior, and lateral walls [10].

The clinical manifestations of vaginal cancer exhibit variability. Predominantly, vaginal bleeding is observed, occurring in approximately 60% of cases. Additional symptoms include alterations in the color and consistency of vaginal discharge (present in 20% of cases), dysuria, rectal pressure, pain during bowel movements, and evolving discomfort in the vaginal and bladder regions as the disease progresses.

Magnetic resonance imaging (MRI) stands as the preferred modality for visualizing intricate vaginal anatomy and pathology. Leveraging exceptional soft tissue contrast and high-resolution multiplanar imaging without ionizing radiation, MRI emerges as an excellent tool for locally staging vaginal and vulvar cancers and monitoring post-treatment outcomes. The integration of positron emission tomography/MR (PET/MR) holds promise for staging and surveillance in gynecologic malignancies, with future investigations expected to elucidate its utility in evaluating vaginal carcinomas [11]. Regrettably, the accessibility of MRI remains limited in Morocco due to its substantial cost, rendering it unavailable to a significant proportion of our patient population.

Considerations for devising a treatment plan for vaginal cancer involve evaluating factors such as the stage, size, and location of the lesion, along with the patient's history of pelvic irradiation and performance status. Primary intervention for invasive disease typically entails a combination of external and intracavitary or interstitial radiotherapy (RT), with surgical options reserved for highly selected early cases that may undergo local excision or brachytherapy alone. Concurrent chemotherapy is frequently administered, drawing from the proven benefits observed in patients with cervix and anus cancers. Favorable outcomes have been reported in a limited number of patients receiving concurrent chemotherapy with RT, aligning with similar approaches in the treatment of anal, cervical, and vulvar cancers. However, randomized controlled trials comparing chemoradiation to radiation alone have not been conducted [1,10,12].

Vaginal cancer has a less favorable outcome compared to both cervical and vulval cancer. Unfavorable prognostic indicators include age over 60, the presence of lesions in the middle and lower thirds of the vagina, and the existence of poorly differentiated tumors in women with this condition.

This study is subject to some limitations, including a small sample size and its retrospective observational, non-interventional design. Despite these constraints, our study provided valuable insights into our clinical practice and enabled comparison with other relevant studies.

## Conclusion

Vaginal cancer, characterized primarily by squamous cell carcinoma, is a rare neoplastic disorder. Diagnosis can be delayed due to patient reticence, emphasizing the importance of heightened vigilance. A comprehensive gynecological examination, including prompt vaginal biopsies when needed, is crucial for accurate diagnosis. Magnetic Resonance Imaging (MRI) aids in characterizing the tumor and assessing its extent. While there is no consensus on the optimal treatment, concurrent radiochemistry is commonly employed. The prognosis is closely tied to the cancer's stage. Ongoing research explores the role of Human Papillomavirus (HPV) in the development of vaginal cancer, highlighting the potential preventive impact of the HPV vaccine. Primary prevention efforts should focus on proactive measures, including prophylactic HPV vaccination.

### What is known about this topic

- Vaginal cancer is a rare malignancy, accounting for 1% - 2% of all female genital tract cancers, with squamous cell carcinoma being the most common histological subtype;
- Risk factors include human papillomavirus (HPV) infection and smoking, although data on HPV prevalence in vaginal cancer remain limited in low- and middle-income countries like Morocco;
- Management of vaginal cancer often involves radiotherapy, chemotherapy, or a combination of both, as surgery is rarely feasible due to the advanced stage at diagnosis.

### What this study adds

- This study provides insights into the management and outcomes of vaginal cancer in a Moroccan cohort, highlighting the challenges posed by delayed diagnosis and socioeconomic constraints;
- It emphasizes the high prevalence of locally advanced disease (T3-T4 stages) and the effectiveness of concurrent chemoradiotherapy in achieving complete remission in nearly 56% of patients;
- The findings underscore the need for improved screening strategies, early detection, and increased access to diagnostic tools such as MRI in resource-limited settings to enhance patient outcomes.
